# Effectiveness of a Nonpneumatic Active Compression Device in Older Adults with Breast Cancer-Related Lymphedema: A Subanalysis of a Randomized Crossover Trial

**DOI:** 10.1089/lrb.2022.0085

**Published:** 2023-12-22

**Authors:** Stanley G. Rockson, Roman Skoracki

**Affiliations:** ^1^ Division of Cardiovascular Medicine, Stanford University School of Medicine, Stanford, California, USA.; ^2^ Department of Plastic Surgery, The Ohio State University Medical Center, Columbus, Ohio, USA.

**Keywords:** breast cancer-related lymphedema, cancer survivorship, quality of life, medical device

## Abstract

**Background:**

A recently completed clinical trial compared a novel nonpneumatic compression device (NPCD) with a traditional advanced pneumatic compression device (APCD) for the treatment of breast cancer-related lymphedema (BCRL); the study revealed that the NPCD produced superior clinical and quality-of-life (QOL) outcomes. In this subanalysis, we sought to examine these results within the subset of trial subjects aged ≥65 years.

**Methods:**

A randomized crossover head-to-head trial was conducted to compare the NPCD with a commercially available APCD. Patients were randomly assigned to one or the other device for 28 days of use, followed by a 4-week washout period before a comparable 28-day utilization of the alternate device. Limb edema, adherence to daily device use, and QOL measures were collected at day 0 and 28 of each period.

**Results:**

A total of 14 subjects were aged ≥65. During NPCD use, subjects experienced a mean decrease in limb edema of 100.3% (*p* = 0.0082) as well as improvements in mean overall and subscale scores of the Lymphedema Quality of Life Questionnaire (LYMQOL). By comparison, during APCD use limb edema decreased by a mean of 2.9% (*p* = 0.8899) with no significant changes in any LYMQOL scores. Mean adherence was significantly higher during NPCD use (96.6%) than during APCD use (58.3%, *p* < 0.0001).

**Conclusions:**

The novel NPCD produced superior clinical and QOL outcomes in older subjects with BCRL. ClinicalTrials.gov ID: NCT04908254

## Introduction

Individuals affected by breast cancer-related lymphedema (BCRL) often experience pain, fatigue, and functional restrictions.^1–6^ As an adjunct to treatment through rigorous self-care regimens of skin care, exercise, manual lymph drainage, and compression garments to reduce limb edema,^7^ the use of pneumatic compression devices may further help to lessen edema and reduce the risk of lymphedema-related complications.^8–14^ Although adherence to BCRL self-care has traditionally been poor,^15–17^ a new technology has shown promise for improving patient adherence and treatment efficacy.

In a recent multicenter randomized crossover head-to-head trial, a novel nonpneumatic compression device (NPCD) produced greater reduction in limb edema and greater improvement in quality of life (QOL) with greater patient adherence and satisfaction than a traditional advanced pneumatic compression device (APCD) in 50 females with BCRL.^18^ In that study, subjects ranging in age from 29 to 84 years experienced an average reduction in limb edema volume of 64.6% with the NPCD, versus 27.7% with the APCD. In addition, mean adherence to the NPCD was 95.6%, with 90% of subjects indicating that they were “somewhat” or “very” satisfied with the device (APCD adherence and satisfaction among the same subjects were 49.8% and 14%, respectively).

Among older breast cancer survivors, the development of lymphedema imposes decrements to QOL on par with that of a major comorbidity.^19^ And, although BCRL prevalence in cancer survivors aged 65+ has been estimated to be 14% within 4 years of cancer treatment,^20^ lymphedema may be substantially underdiagnosed in this population.^21^ Given that physical activity can reduce health care utilization and costs in the general population of older adults^22,23^ and that exercise has been linked to improved BCRL outcomes,^24^ the use of a compression device that allows for continued movement and activity during use may be of particular importance for older BCRL patients. Since the NPCD was designed to allow for such movement and activity (in contrast to traditional APCDs that require patients to be immobile during treatment), we sought to determine whether the meaningful clinical superiority of the NPCD observed in the full trial cohort^18^ would be replicated in the subpopulation of trial subjects ≥65 years.

## Methods

**FIG. 1.**
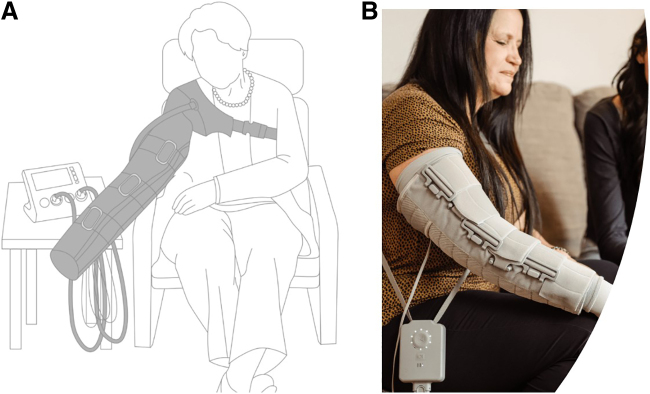
The commercially available advanced pneumatic compression device **(A)** and the novel nonpneumatic compression device **(B)**. Image used with subject's permission.

Details of the trial are presented elsewhere,^18^ but, in brief, unilateral BCRL subjects were recruited and randomized to either the NPCD or a commercially available APCD (Fig. 1) for 28 days, followed by a 4-week washout period without any device use, at which time they crossed over to the alternate device for another 28 days. Limb volume was calculated for both arms using truncated cone cylindrical segment analysis, and the difference in volume between the affected and unaffected arm was used as an index of edema.

The current analysis is limited to the 14 trial subjects aged ≥65 years (at study initiation). Changes in edema volume and QOL (using the Lymphedema Quality of Life Questionnaire, or LYMQOL)^25^ from day 0 to 28 of each device use period were measured and compared. The LYMQOL includes four subscales (Function, Appearance, Symptoms, and Mood) where lower values reflect less impairment, and an overall QOL score where higher values reflect better QOL. We examined both the absolute and percentage change in edema volume; responders were defined as patients whose edema volume at day 28 was reduced from day 0. Adherence reflected the percentage of total prescribed hours used as self-reported by subjects. In addition, change in edema volume by adherence was examined using Pearson correlations and linear regression. Statistical analyses included McNemar's chi-squared test for categorical variables and paired *t*-tests for continuous variables.

The study was approved by the WCG Institutional Review Board, and patients provided informed consent to participate.

## Results

Among the 14 subjects aged ≥65, the mean age was 72.5 years (max = 84 years); 9 were Caucasian, 1 African American, 2 Asian, and 2 Hispanic. During NPCD use, the mean percentage change in edema was a decrease of 100.3% (95% confidence interval [CI] 30.8–169.7 decrease, *p* = 0.0082, Table 1). During APCD use, the mean change was a decrease of 2.9% (95% CI −47.1 to 41.3, *p* = 0.8899). The percentage reduction in edema experienced during NPCD use was significantly greater than during APCD use (mean difference = 97.4%, *p* = 0.0034).

**Table 1.** Changes in Edema from Day 0 to 28 Among Subjects Aged 65 Years and Older (*N* = 14)

	NPCD	APCD
Total *N*	14	14
Responders, *n* (%)	12 (85.7%)	1 (7.1%)
*p*-Value NPCD versus APCD	0.003	
Absolute edema change^a^ in the affected arm, mean cm^3^	−259.9	+100.2
*p*-Values for change significantly different from 0	0.001	0.039
*p*-Value for NPCD versus APCD	0.000	
Percentage edema change^a^ in the affected arm, mean cm^3^	−100.3%	−2.9%
*p*-Values for change significantly different from 0	0.008	0.890
*p*-Value for NPCD versus APCD	0.003	

^a^ From day 0 to 28; a negative change means a reduction in edema.

APCD, advanced pneumatic compression device; NPCD, nonpneumatic compression device.

Furthermore, subjects experienced significant improvements in mean LYMQOL subscale scores (Function, Appearance, Symptoms, and Mood) and Overall QOL (all *p* < 0.01; Table 2) during NPCD use. No improvement in LYMQOL scores occurred during APCD use.

**Table 2.** Means and Changes in Lymphedema Quality of Life Questionnaire Scores Among Subjects Aged 65 Years and Older (*N* = 14)

Day 0	NPCD			APCD			
	Mean	SD		Mean	SD		
Function	2.0	0.5		1.6	0.5		
Appearance	2.5	0.7		1.8	0.6		
Symptoms	2.5	0.8		2.1	0.7		
Mood	2.1	0.7		1.8	0.5		
Overall QoL^a^	5.6	2.0		7.0	1.5		

Day 28	Mean	SD		Mean	SD		
Function	1.4	0.4		1.8	0.7		
Appearance	1.8	0.7		2.2	0.9		
Symptoms	1.8	0.7		2.3	0.7		
Mood	1.4	0.4		2.0	0.8		
Overall QoL	8.4	1.3		6.2	2.2		

Change (day 28–0)	Mean	SD	*p* ^b^	Mean	SD	*p* ^b^	*p* ^c^
Function	−0.6	0.6	0.002	0.3	0.4	0.022	0.000
Appearance	−0.7	0.5	0.000	0.4	0.7	0.029	0.001
Symptoms	−0.7	0.6	0.001	0.2	0.6	0.284	0.010
Mood	−0.7	0.6	0.001	0.2	0.5	0.079	0.002
Overall QoL^a^	2.9	2.1	0.000	−0.5	2.1	0.375	0.002

Lower values for individual domains mean less impairment (better); higher values for Overall QoL reflects higher QoL (better).

^a^ *N* = 13 for APCD.

^b^ Change from day 0 to 28 within device.

^c^ Difference between NPCD and APCD.

LYMQOL, lymphedema quality of life questionnaire; QoL, quality of life; SD, standard deviation.

Mean adherence for the NPCD was 96.6% compared with 58.3% of the APCD (*p* < 0.0001). On average, subjects used the NPCD longer (a mean of 28.3 hours over the 28-day period, or >1 h/day) than they did the APCD (mean of 16.3 hours, or ∼35 min/day; mean difference in total hours = 12.0, *p* < 0.0001). When asked, 11 of 14 (78.6%) indicated that their use of compression sleeves lessened during NPCD treatment, compared with 0% during APCD use. Patients also indicated that the NPCD facilitated exercise and was convenient for travel.

Because adherence to the NPCD was universally high, examinations of the association between adherence and change in edema was limited to the APCD. The correlation between adherence and edema change was positive but not significant for both absolute change (correlation = 0.38, *p* = 0.1784) and percentage change (correlation = 0.21, *p* = 0.4773). When absolute edema change during APCD use was plotted against adherence to the APCD, a positive but nonsignificant coefficient was estimated for adherence, suggesting that there were no statistically significant differences in absolute edema change by adherence level for the APCD (data not shown). Similar results were found when percentage edema change was plotted against adherence.

## Discussion

The results indicate that the clinical and QOL benefits of the NPCD observed in the original RCT are replicated within the subgroup of subjects ≥65. Specifically, the NPCD resulted in significantly greater edema reduction and improvements in LYMQOL scores within this subset. In addition, subjects were significantly more adherent to the NPCD than to the APCD, and indicated that the NPCD facilitated exercise and was convenient for travel.

Subjects used the NPCD for almost twice as long per day than the APCD, on average. Although speculative, this could reflect the fact that the NPCD is more comfortable and conducive to prolonged wear. Although the sequential compression mechanisms employed by the two devices are similar, the NPCD also provides a base static compression that is not available with APCD and allows movement/mobility during use. Although the improvements in edema reduction are correlated to the frequency and duration of use, the additional mechanism of static compression and mobility/movement could have further aided in the improvement observed.

In addition to greater mobility, subjects indicated that the NPCD facilitated exercise, which has been linked to better outcomes in BCRL patients.^24^ Although previous research has demonstrated the clinical benefits and reduced health care utilization associated with the use of compression devices,^13,14^ the need to remain immobile during treatment with traditional pneumatic devices may limit exercise. Although the results of this study demonstrate the superiority of the NPCD over a 28-day period, we acknowledge that this is a relatively short follow-up period.

In addition, the novelty of the NPCD may have been attractive for some patients, which could have resulted in higher adherence over the study period. The original study was not powered for a subanalysis, and the results should be viewed with caution, given that the sample size is 14. Finally, we did not adjust our threshold for statistical significance to account for multiple comparisons; however, many of the *p*-values were highly significant and likely would remain statistically significant after adjustment. The novel NPCD allows older adults to maintain mobility during use while significantly reducing limb edema and improving QOL.
